# Study of Botany by Young Physicians

**Published:** 1845-03

**Authors:** 


STUDY OF BOTANY BY YOUNG PHYSICIANS.

We seize the occasion afforded by the publication, in this number, of an article on the Flora of Illinois, from our respected colleague, Professor Short, to commend the study of Botany to the young physicians of the West. After traveling, within the last three years, nearly 15,000 miles, between and on the Lakes and the Gulf of Mexico, we are able to recollect (out of Louisville) the following physicians only, who cultivate that science. Dr. Carpenter, of New Orleans, one of the Professors of the Medical College of Louisiana, who at the same time attends to other branches of physical science; Dr. Hale, of Alexandria, Louisiana; Dr. Engelmann, of St. Louis, whose communications in relation to some of the objects of this science, have, we understand, been published in Europe, and elicited a complimentary notice from the highest authorities; Dr. Mead, of Illinois; Dr. Chapman, of Quincy, Florida, from whom we have reason, soon, to expect a systematic catalogue of the plants of that El Dorado of American Botanists; Dr. Peter, now Professor of Chemistry in Transylvania University; Dr. Clapp, of New Albany, Indiana; Dr. Bigelow, of Lancaster, Ohio, who has published a local catalogue; Dr. Warder, of Cincinnati, whose city resi dence, professional duties, and taste for other branches of natural history, are, however, diverting him from the study of plants; Dr. Houghton, of Michigan, its chief geologist, and a professor in its University, who, to medicine, has added the cultivation of botany geology, and mineralogy; lastly, Dr. Riddell, formerly of Ohio, and author of a catalogue of its plants, now, however, a professor of Chemistry in New Orleans, and Refiner of the Mint. Thus, he has abandoned the gardens of *Flora* for the smithies of *Plutus!* When we saw him last spring, instead of being engaged in arranging and describing an herbarium of rare plants, which he had brought from the interior of Texas, two or three years before, his spare time was diligently devoted to the preparation of a book of fac similies of all the spurious coins he could gather together! A collector of flowers transformed into a collector of counterfeit dollars! Well was it for the goddess, that she died off 1800 years ago—happy for her, that she did not live to witness the backsliding of one of her most ardent and gifted sons. To come nearer home with this criticism; we are obliged to state, that Dr. Clapp, after many years of diligent research as a local botanist, has, also, been diverted from the study, but in a different direction from that of Dr. Riddell. Enamoured with the organic remains, and especially the fossil corals, with which the rocks of this neighborhood abound, he has given up his leisure time to *their* study; and with a zeal, rare, indeed, in middle life, and therefore the more admirable, has even studied the German language, for the sole purpose of reading a single distinguished, untranslated work on that branch of Natural History! It must not be said, that these pursuits have been at the expense of his profession; for, in both its literature and practice, he 'keeps up with the times.' And so might *hundreds* of our brethren, if they had the same industry and love of science. But we must come closer to our main object.

Why is it, that in the Great Valley and its borders, all parts of which abound in native plants, not a dozen physicians, cultivate the science of Botany? Does it indicate, that they have maturely investigated and rejected its claims upon them? We trow not. Most of them have given so little attention to the subject, that they cannot even tell, whether that which at any time strikes their eye, is a 'botanical plant' or not. No, the truth is, they cannot so much as pretend that they have intelligently decided whether they ought to study botany. But, perhaps, their leisure time is given up to other collateral and auxiliary branches of science:—to geology, as disclosing the composition of the surface of the earth, and the waters it spouts up—thus illustrating the probable origin of certain diseases of their neighborhood—to zoology and comparative anatomy—to systematic and diversified meteorological observations—to post-mortem inspections and pathological anatomy! No, such is not the fact. These departments are as little cultivated as botany. In a spirit of rigid justice, the strictest impartiality is observed—and no branch can boastfully say to the rest, *I* am the favorite. Again, however, we must return to the proper subject of our homily.

What, then, are the reasons, that so few of our younger brethren cultivate botany? They appear to be chiefly the three following—1st. The want of studious habits. 2d. The want of scientific taste. 3d. The want of classical learning. Now, we can give a prescription for the whole of these wants, which, if taken, will be infallible—it is, at once to enter on the study of the science, whose claims we are asserting. The collection and examination of plants will promote industry, inspire a love of nature, and prompt to the study of the Latin language. By the end of the first year after graduation, all will be easy. An hour in the twenty-four devoted to the examination and preservation of the plants, collected in visiting a patient, and another dedicated to the Latin grammar and dictionary, would, after deducting eight for sleep, leave fourteen for other reading and attendance on the sick—several more, we apprehend, than are usually occupied. Such are the easy terms, on which all that we recommend, might be accomplished. Now what are the special motives for pursuing this course? We will enumerate a few.

1st. Every physician ought to have some knowledge of the learned languages; but when he has not studied them at school, he will not do it after graduating, unless he have a particular object in view. The cultivation of Botany would be such an object. Moreover, most of those who do acquire some knowledge of those languages, before they engage in the study of medicine, lose it in a few years; but the study of Botany would not only preserve, but extend it.

2d. The Valley of the Mississippi is not, like Europe, exhausted of its indigenous vegetation, but abounds in native plants, many of which are peculiar to it, and interesting for their beauty or singularity.

3d. Its different latitudes and soils present nearly all the medicinal plants of the United States, not a few of which are deserving of more attention than they have received, and ought to be made subjects of pharmaceutic and clinical experiment by our physicians, as they undoubtedly would be, if botany were studied.

4th. Botany is a science of visible relations—and its study is eminently fitted to improve the faculty of observation.

5. The chemico-vital and vegetative functions of animals and plants, as developed in these latter days, have so many common characters or close analogies, that no physiologist can be either profound or comprehensive in his knowledge, unless it embrace the latter as well as the former, a new motive for adding botany to his other studies.

6th. Without an acquaintance with the elements of Botany, even a common dispensatory is in part a sealed book to the physician; but such ought not to be the case. When he sees at the beginning of an article the words, *Datura Stramonium*—Pentandria Monogynia—Nat. Ord., Solaneae, he recognizes in the first a name, but, if ignorant of the science, is unable to tell his student, or realize to himself, that the other words are full of meaning, both as to the forms and qualities of the plant.

7th. The physician who practises in the country, would find an interest in the flowers which smile along his path, well calculated to relieve the *tedium* of his solitary rides; and this pleasure would sometimes reward him, for labors which might bring no other compensation.

Students and young physicians are often deterred from entering on the study of botany, by its repulsive nomenclature, and the immense number of its subjects; but there is no science in which they are sooner overcome—no temple in which the difficulties are so much congregated in the vestibule, as that of Flora. A great number of the hard words spring from the same roots, and are but modified multiples of each other. Moreover, most of them are words with which every man belonging to the profession of medicine, ought to be acquainted, independently of their use in botany. Then, again, as to the multitude of plants, they constitute natural groups or families, the different members of which, have so many characters in common, that when one has been studied, all the rest become quite easy. Take, as an example, the genus *Quercus*, or oak. When the flower and fruit of one species are known, it only remains to compare the leaves of trees having the same fructification, to make out the thirty-two species, which Nuttall tells us are indigenous to the United States.

Our limits do not admit of other suggestions. We look forward with much interest to the publication of Professor Short's Flora of the Mississippi Valley. His collections, the labor of a quarter of a century or more, are of vast extent; and we cannot but cherish the hope, than when his book shall appear, it will give an impulse to the study of botany throughout the extended region, from which its materials have been drawn; and upon which we cannot doubt it will confer scientific distinction. D.

